# 

**Published:** 1908-10-03

**Authors:** 


					T?l.graphio AddreaaSf ^ - tfl'THE MODERN T.Uphone Number i
Q.p?d?i,. London. MEDICAL JOURNAL. 2734 Gerrard.
/T.TP.TJA'PY
"EW n"'iE5S4. v0?i8xlvV0,-,V' SURG&MJ?fcfS5
RISKS. ffiffflCa908.
PRICE THREEPENCE

				

